# Impact of chemoradiotherapy for first primary lung cancer on the prognosis and re-chemoradiotherapy sensitivity of second primary lung cancer

**DOI:** 10.3389/fimmu.2025.1492501

**Published:** 2025-01-27

**Authors:** Zhe Chen, Gaoming Wang, Nan Wang, Jiangjiang Liu, Yu Yao, Haitao Ma, Jing Luo, Kai Xie

**Affiliations:** ^1^ Department of Cardiothoracic Surgery, The Fourth Affiliated Hospital of Soochow University, Suzhou, China; ^2^ Department of Thoracic Surgery, Xuzhou Central Hospital, Clinical School of Xuzhou Medical University, Xuzhou, China; ^3^ Department of Respiratory Medicine, Nanjing Second Hospital, Nanjing University of Chinese Medicine, Nanjing, China; ^4^ Department of Thoracic Surgery, The First Affiliated Hospital of Soochow University, Suzhou, China; ^5^ Department of Cardiothoracic Surgery, Jinling Hospital, Medical School of Nanjing University, Nanjing, China

**Keywords:** radiotherapy, chemotherapy, second primary lung cancer, re-chemoradiotherapy sensitivity, prognosis

## Abstract

**Background:**

Despite undergoing surgery and chemoradiotherapy, patients with first primary lung cancer (FPLC) remain at risk for second primary lung cancer (SPLC), which is associated with a poor prognosis. The effects of FPLC chemoradiotherapy on SPLC prognosis and its sensitivity to re-chemoradiotherapy have not been adequately investigated.

**Methods:**

This cohort study analyzed data from 23,827 patients who underwent FPLC surgery during 1973–2021, drawn from the Surveillance, Epidemiology, and End Results database. Among these, 5,302 FPLC patients developed SPLC within 5 years of their initial diagnosis. We employed the Fine-Gray competitive risk model, Cox proportional hazards model, and restricted mean survival time analysis to assess the effects of FPLC radiotherapy and chemotherapy on SPLC risk and survival differences.

**Results:**

The competitive risk model indicated that FPLC radiotherapy and chemotherapy did not significantly change the risk of developing SPLC. However, the Cox proportional hazards model revealed that FPLC radiotherapy was associated with decreased overall survival (OS; HR=1.251, P<0.001) and cancer-specific survival (CSS; HR=1.228, P=0.001) in patients with SPLC. Conversely, FPLC chemotherapy was linked to improved OS (HR=0.881, P=0.012) in this population. Patients with SPLC who received combined chemoradiotherapy for FPLC exhibited significantly reduced survival times (OS: HR=1.157, P=0.030; CSS: HR=1.198, P=0.018), a finding confirmed across multiple models. For SPLC patients with prior FPLC chemoradiotherapy, subsequent SPLC radiotherapy significantly improved prognosis. Notably, this benefit is even more pronounced in patients who have not received prior chemoradiotherapy. While SPLC chemotherapy enhanced OS for patients who did not receive FPLC chemotherapy, it was associated with reduced CSS for those who had.

**Conclusions:**

Overall, FPLC chemoradiotherapy influences SPLC prognosis and influences sensitivity to treatment. Tailoring SPLC management to FPLC treatment regimens may improve survival outcomes.

## 1 Introduction

Patients with resected tumors often undergo radiotherapy or chemotherapy to improve their prognosis (1, 2). However, these treatments can lead to adverse side effects and may increase the risk of developing second primary tumors. For example, radiotherapy for colorectal cancer is associated with a heightened risk of second primary gynecological tumors (3), while high-dose chemotherapy is linked to an increased incidence of non-solid tumors (4). Additionally, recent literature indicates that the response to subsequent malignancy treatment varies depending on prior treatment exposures, which is critical for survival and disease management. For example, radiation therapy can restore immune activity in non-small cell lung cancer that has developed resistance to checkpoint inhibitors (5), while patients with small-cell lung cancer who receive first-line immunotherapy plus chemotherapy show a favorable response to carboplatin-etoposide treatment (6). Therefore, strategies aimed at enhancing survival through radiotherapy or chemotherapy in primary tumors must be thoughtfully applied to the management of second primary tumors.

Patients with first primary lung cancer (FPLC) face a continuous risk of developing second primary lung cancer (SPLC). Annually, 1%-2% of these patients receive a diagnosis of a new lung cancer following the surgical resection of FPLC (7). SPLC is associated with a higher mortality rate compared to FPLC (8). The definition of SPLC following FPLC varies; however, a common threshold is a 2-year latency period proposed by Martini et al. (9). Other studies have utilized latency periods of 3, 4, and 5 years to define SPLC (10–12); however, the characteristics of these varying definitions, including differential risk factors and treatment sensitivities, remain insufficiently explored.

Radiotherapy and chemotherapy, which target DNA replication, may impart distinct biological traits to new SPLCs. Particularly, radiochemotherapy may heighten the risk of genetic mutations, promoting the emergence of diverse cancer phenotypes (13). For instance, radiation therapy can generate unique mutational signatures in second cancers (14), and the use of platinum-based chemotherapy is associated with an increase in partial chromosomal copy number in secondary malignancies (15). Although the association between radiotherapy for FPLC and SPLC prognosis is documented (16), the relationship involving FPLC chemotherapy or combination therapy is less clear. Observations indicate that the prognosis for patients with second tumors who previously received radiotherapy differs markedly from those without such history (3), underscoring the need for further research into the prognostic implications of FPLC treatments for SPLC.

While previous studies have identified the risk of SPLC among various FPLC populations (12), these studies have not specifically explored how a history of chemoradiotherapy for FPLC influences SPLC prognosis or the survival benefits of SPLC treatment. Most existing research has focused on the impact of FPLC treatments on the incidence of SPLC, while the long-term survival benefits for patients with SPLC who have a history of chemoradiotherapy for FPLC remain underexplored. This gap in knowledge forms the basis for our study. The primary motivation for this research is to examine how FPLC chemoradiotherapy history impacts the incidence and prognosis of SPLC. By focusing on the long-term outcomes of patients who have undergone chemoradiotherapy for FPLC, our study aims to provide clinically relevant insights into the potential survival benefits of subsequent chemoradiotherapy in SPLC treatment. The findings from this study will contribute to a better understanding of the clinical management of this patient population and enhance the practical application of treatment strategies in the context of both FPLC and SPLC.

## 2 Materials and methods

### 2.1 Database and participants

**Figure 1:**
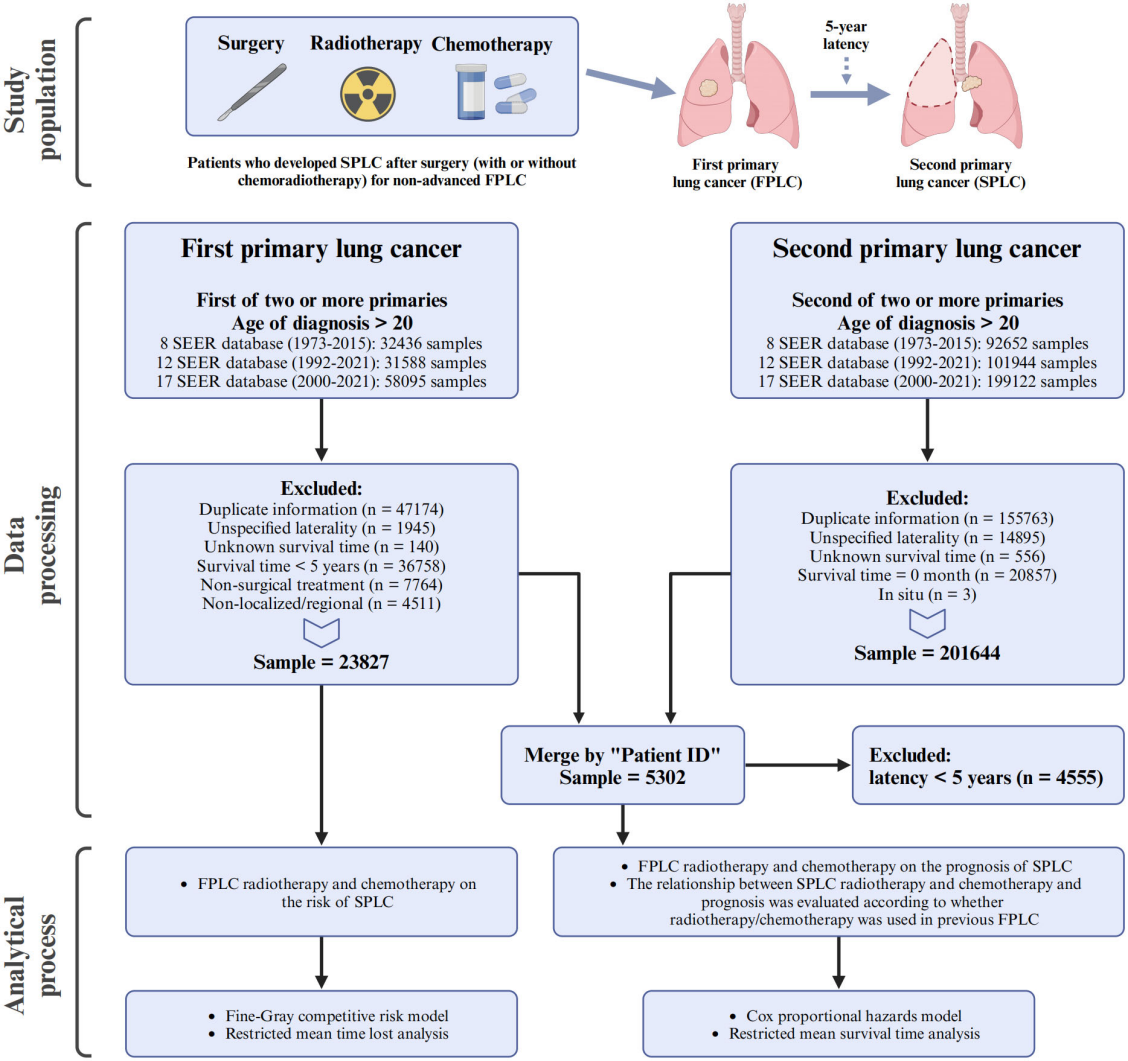
Flowchart of the study design.

Patients with lung cancer, including both FPLC and SPLC, were identified from the Surveillance, Epidemiology, and End Results (SEER) database, adhering to the third edition of the International Classification of Diseases for Oncology (C34.0–C34.9) and utilizing local, regional, and distant staging systems. The patient screening process is illustrated in **Figure 1**. For the FPLC cohort, patients were included if they were diagnosed with FPLC as the first of two or more primary cancers, were older than 20 years at diagnosis, and were identified from three SEER datasets spanning 1973–2021. FPLC patients were excluded for the following reasons: duplicate information (n = 47174), unspecified laterality (n = 1945), unknown survival time (n = 140), survival time less than 5 years (n = 36758), non-surgical treatment (n = 7764), or non-localized/regional stage (n = 4511). After these exclusions, the final FPLC cohort included 23827 patients. For the SPLC cohort, patients were included if they were diagnosed with SPLC as the second of two or more primary cancers, were older than 20 years at diagnosis, and were identified from the same SEER datasets. Exclusions were made for duplicate information (n = 155763), unspecified laterality (n = 14895), unknown survival time (n = 556), survival time of 0 months (n = 20857), or *in situ* carcinoma (n = 3), resulting in a final sample size of 201644 SPLC patients.

During the screening process, patients with unknown laterality and unknown survival time were removed to ensure data completeness. In the FPLC cohort, these exclusions accounted for approximately 2.2% of the initially included population, while in the SPLC cohort, they accounted for approximately 3.8%. These exclusions are consistent with standard practices for handling incomplete data in SEER database studies. Given the small proportion of excluded cases, the impact of these exclusions on the dataset’s completeness and the reliability of subsequent analyses is relatively small.

The two cohorts were then merged by “Patient ID,” and patients with a latency of less than 5 years between FPLC and SPLC diagnoses (n = 4555) were further excluded. After these steps, a final dataset comprising 5302 patients was established and used for subsequent analyses.

As the SEER database operates as a multi-center, open-registry system for cancer patients, this study was exempt from the need for patient consent or ethical approval, thereby enhancing its generalizability to real-world scenarios. This retrospective study has been reported in accordance with the STROCSS criteria (17).

### 2.2 Definition and follow-up of SPLC

In this study, based on previous reports, SPLC was defined as a malignant lung tumor occurring 5 years or more after the diagnosis of FPLC (12, 18). To refine this definition and ensure a clear distinction from recurrent disease, we excluded any FPLC patients with distant metastases (TNM staging M1), as these cases could potentially confound the identification of true SPLC cases. According to the SEER database, surgical interventions in these patients are defined as “removal of all cancerous areas visible to the naked eye”. Furthermore, patients’ records with a “Sequence number” indicating “2nd of 2 or more primaries” adhere to ICD-O-3 guidelines, effectively excluding recurrent diseases (19). This approach ensures that our FPLC cohort consisted of non-advanced stage patients who were amenable to surgical intervention, thereby minimizing the influence of recurrent FPLC on the definition and subsequent analysis of SPLC. The primary aim of this study was to assess the 10-year survival of SPLC, focusing on overall survival (OS) and cancer-specific survival (CSS), measured from the time of SPLC diagnosis. OS was defined as death from any cause during follow-up, while CSS was defined as death specifically attributable to the tumor.

### 2.3 Propensity score matching

PSM was employed to control for bias due to baseline differences. Logistic regression balanced clinical characteristics between FPLC and SPLC across different subject groups. Untreated and treated groups were assigned values of 0 and 1, respectively, with a 1:1 matching ratio applied using a caliper value of 0.1. The effectiveness of this matching process was evaluated through the standardized mean difference (SMD) and the significance of the chi-square test for baseline variables in the matched groups. An SMD of less than 0.1 and a chi-square test P-value of less than 0.05 indicated favorable pairing.

### 2.4 Statistical analysis

Statistical analyses included the Mann-Whitney U test for comparing two independent groups of continuous variables, while the Kruskal-Wallis test was applied for comparisons involving more than two groups. For categorical variables, the Chi-squared test was utilized. The association between FPLC chemoradiotherapy and the risk of developing SPLC was evaluated using the Fine-Gray competing risks model, with all-cause mortality considered as a competing event. Additionally, the Cox proportional hazards model was employed to investigate the influence of study factors on prognosis. Hazard ratios (HRs) were calculated to assess the effectiveness of these factors in the models, highlighting multifactorial analysis as the principal finding. Moreover, the study conducted analyses using the restricted mean time lost (RMTL) and restricted mean survival time (RMST), which estimate differences in the area under the time-to-event curves (RMTLd: the difference in restricted mean time lost; RMSTd: restricted mean survival time difference) without relying on the proportional hazard assumption. The consistency observed between Fine-Gray and RMTL analyses, as well as between Cox and RMST analyses, suggests a minimal likelihood of bias from violations of the proportional hazard assumption, and offering additional perspectives on the average time of event occurrence in different group. The characteristics of these four statistical methods are detailed in **Supplementary Figure S1**. Significant predictors identified in univariate analysis, along with the characteristics of chemoradiotherapy in FPLC, were incorporated into the multivariate models. Subgroup analyses based on clinical features of FPLC and SPLC were performed to calculate interaction effects between characteristics of subgroups and the primary objectives of the study. Statistical significance was determined at *P*-values less than 0.05. All statistical analyses were conducted using R software, version 4.3.3.

## 3 Results

### 3.1 Study design

The study design and population inclusion and exclusion criteria are shown in **Figure 1**. We adopted a comprehensive research approach with two main objectives: assessing the effect of FPLC chemoradiotherapy history on prognosis and the sensitivity of SPLC to re-chemoradiotherapy.

### 3.2 Patient characteristics

Initially, 23,827 patients with non-advanced FPLC who underwent surgical resection were considered for the study, with their baseline characteristics detailed in **Supplementary Tables S1**–**S3**. After matching FPLC patients with SPLC cases and excluding those with missing data, 5,302 SPLC patients were analyzed (**Supplementary Figure S1**). For SPLC patients, the median age at diagnosis was 64 years (interquartile range [IQR] 58-70) for FPLC and 73 years (IQR 67-79) for SPLC. The median latency from FPLC diagnosis to SPLC diagnosis was 97 months (IQR 75-132) for both groups. During the FPLC treatment period, 594 patients (11.203%) received radiotherapy, 958 (18.069%) underwent chemotherapy, and 370 (6.978%) received both treatments. The baseline characteristics of SPLC patients are presented in **Supplementary Tables S4**–**S6**.

### 3.3 Risk of SPLC attributable to FPLC chemoradiotherapy

Baseline FPLC data were used to assess the impact of FPLC therapy on the risk of developing SPLC. Multivariate analysis indicated that radiotherapy, chemotherapy, and chemoradiotherapy for FPLC were not statistically linked to an increased risk of SPLC compared to the control group. Additionally, no significant differences in SPLC risk were observed concerning FPLC radiotherapy, chemotherapy, or chemoradiotherapy across various treatment groups (**Table 1**; **Supplementary Tables S7**, **S8**). Consistent findings were obtained in RMTL analysis adjusted for covariates (**Supplementary Table S9**).

**Table 1:** Risk of developing SPLC in patients with different therapies for FPLC.

Variables	SPLC risk			
	Unadjusted HR (95%CI)	*P value*	Adjusted HR (95%CI)	*P value*
Radiotherapy of FPLC				
No	1.000 (Reference)		1.000 (Reference)	
Yes	1.026 (0.943 ~ 1.116)	0.550	1.031 (0.939 ~ 1.131)	0.520
Chemotherapy of FPLC				
No	1.000 (Reference)		1.000 (Reference)	
Yes	1.102 (1.029 ~ 1.181)	0.006	0.999 (0.924 ~ 1.079)	0.980
Chemoradiotherapy of FPLC				
No chemotherapy and radiotherapy	1.000 (Reference)		1.000 (Reference)	
Only chemotherapy	1.094 (1.004 ~ 1.191)	0.040	0.977 (0.895 ~ 1.067)	0.610
Only radiotherapy	0.940 (0.823 ~ 1.074)	0.360	0.978 (0.854 ~ 1.120)	0.750
Chemotherapy and radiotherapy	1.107 (0.997 ~ 1.229)	0.057	1.059 (0.952 ~ 1.178)	0.290
Chemoradiotherapy of FPLC*				
Only chemotherapy	1.000 (Reference)		1.000 (Reference)	
Only radiotherapy	0.860 (0.738 ~ 1.001)	0.051	1.001 (0.856 ~ 1.170)	0.990
Chemotherapy and radiotherapy	1.012 (0.890 ~ 1.150)	0.860	1.083 (0.952 ~ 1.233)	0.230
Chemoradiotherapy of FPLC**				
Only radiotherapy	1.000 (Reference)		1.000 (Reference)	
Chemotherapy and radiotherapy	1.177 (0.999 ~ 1.387)	0.051	1.082 (0.917 ~ 1.277)	0.084

Fine-Gray competitive risk model was used to calculate the HRs and 95%CIs of SPLC risk in FPLC patients receiving different treatments. Controls were replaced under the same analysis, * indicates that the reference group was only FPLC chemotherapy, and ** indicates that the reference group was only FPLC radiotherapy.

### 3.4 Survival of SPLC attributable to FPLC chemoradiotherapy

**Figure 2:**
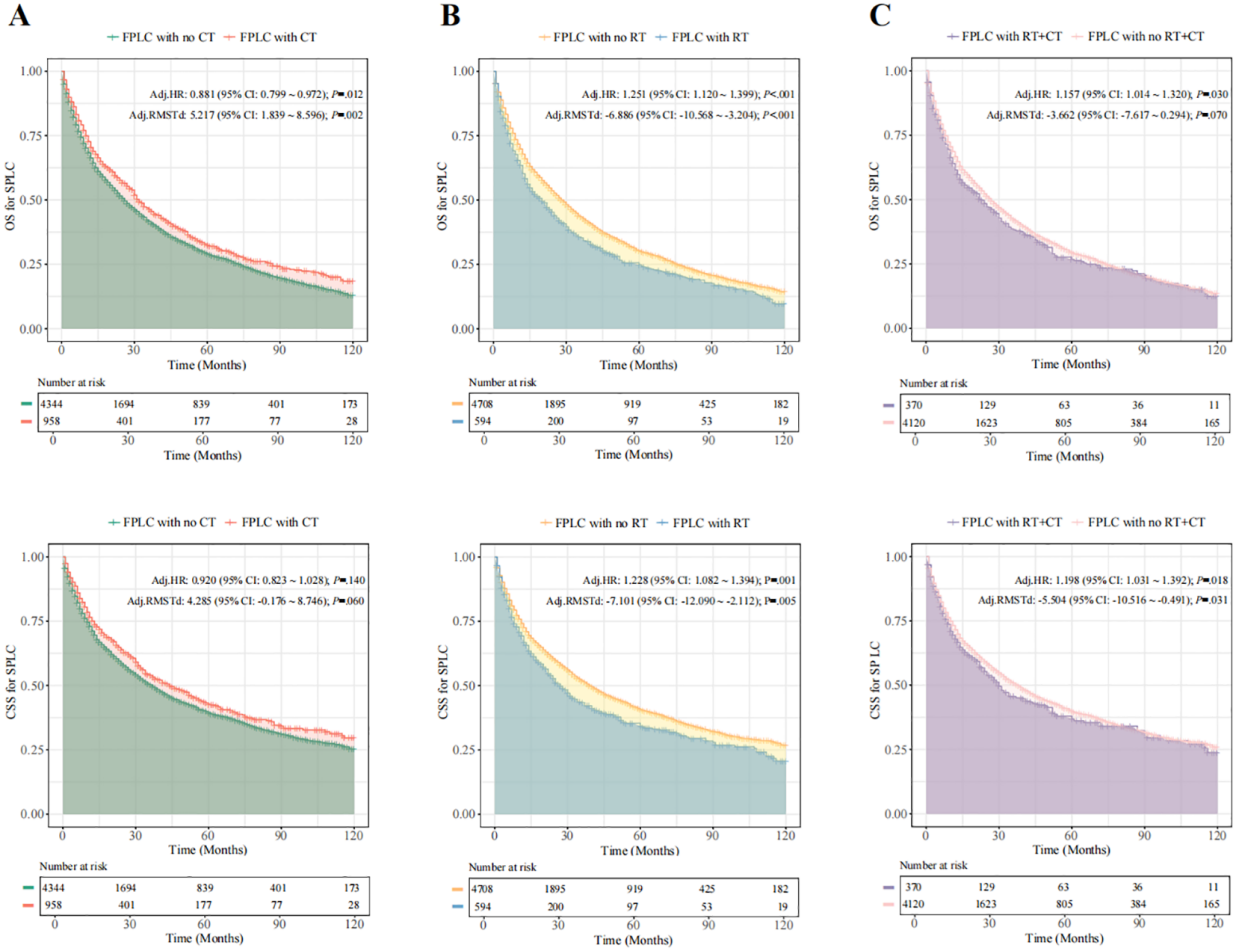
SPLC-related survival in patients with different therapies for FPLC. Fine-Gray competitive risk model was used to calculate the HR and 95% CI. **(A)**, SPLC-related survival in patients with and without FLC radiotherapy for FPLC. **(B)**, SPLC-related survival in patients with and without FLC chemotherapy for FPLC. **(C)**, SPLC-related survival in patients with and without FLC chemoradiotherapy for FPLC. FPLC, first primary lung cancer; SPLC, second primary lung cancer; HR, hazard ratio; RMSTd, restricted mean survival time difference; CI, confidence interval; OS, overall survival; CSS, cancer-specific survival; RT, radiotherapy; CT, chemotherapy.

When comparing the prognostic outcomes for SPLC patients with and without a history of FPLC chemoradiotherapy, adjusted Cox analysis revealed that patients with a history of FPLC radiotherapy exhibited significantly lower overall survival (OS) and cancer-specific survival (CSS) than those without such a history (OS: HR = 1.251, 95% CI = 1.120-1.399, *P* < 0.001; CSS: HR = 1.228, 95% CI = 1.082-1.394, *P* = 0.001). Conversely, a history of FPLC chemotherapy demonstrated a protective effect on the OS of SPLC patients (OS: HR = 0.881, 95% CI = 0.799-0.972, *P* = 0.012; CSS: HR = 0.920, 95% CI = 0.823-1.028, *P* = 0.140). SPLC patients with a history of both FPLC radiotherapy and chemotherapy also experienced lower OS and CSS compared with the control group (OS: HR = 1.157, 95% CI = 1.014-1.320, *P* = 0.030; CSS: HR = 1.198, 95% CI = 1.031-1.392, *P* = 0.018). The mean OS time for SPLC patients with a history of FPLC chemoradiotherapy did not show a significant difference (RMSTd = -3.662 months, 95% CI = -7.617-0.294, *P* = 0.070). After adjusting for covariates, RMST analysis results corroborated the findings of the multivariable Cox regression (**Figure 2**; **Supplementary Tables S10**–**S13**).

### 3.5 Prognostic differences in multiple models

To evaluate the robustness of the impact of FPLC radiotherapy and chemotherapy history on SPLC prognosis, we constructed multiple models to test this hypothesis. The Cox regression analysis was adjusted for characteristics of both FPLC and SPLC. When adjusted solely for SPLC characteristics, the conclusions aligned with the initial model, suggesting that the influence of FPLC recurrence on SPLC survival is relatively minor (**Supplementary Tables S10**, **S12**). PSM was employed to further mitigate confounding bias. After matching, we included 506 patients with a history of radiotherapy, 750 with a history of chemotherapy, and 337 with a history of chemoradiotherapy. Differences in baseline characteristics between the treated and untreated groups were not statistically significant, with all standardized mean differences (SMD) below 0.1 (**Supplementary Tables S14**–**16**). The adjusted model post-PSM continued to indicate that a history of FPLC radiotherapy was associated with poorer prognosis for SPLC (OS: HR = 1.260, 95% CI = 1.089-1.459, *P* = 0.002; CSS: HR = 1.192, 95% CI = 1.010-1.407, *P* = 0.038), while FPLC chemoradiotherapy history was associated only with reduced OS in SPLC patients (OS: HR = 1.227, 95% CI = 1.020-1.476, *P* = 0.030; CSS: HR = 1.129, 95% CI = 0.919-1.388, *P* = 0.246). The matched data did not reveal any effect of FPLC chemotherapy history on the survival of SPLC patients (OS: HR = 0.896, 95% CI = 0.789-1.018, *P* = 0.091; CSS: HR = 0.919, 95% CI = 0.795-1.062, *P* = 0.250; **Supplementary Tables S17**–**S19**).

**Figure 3:**
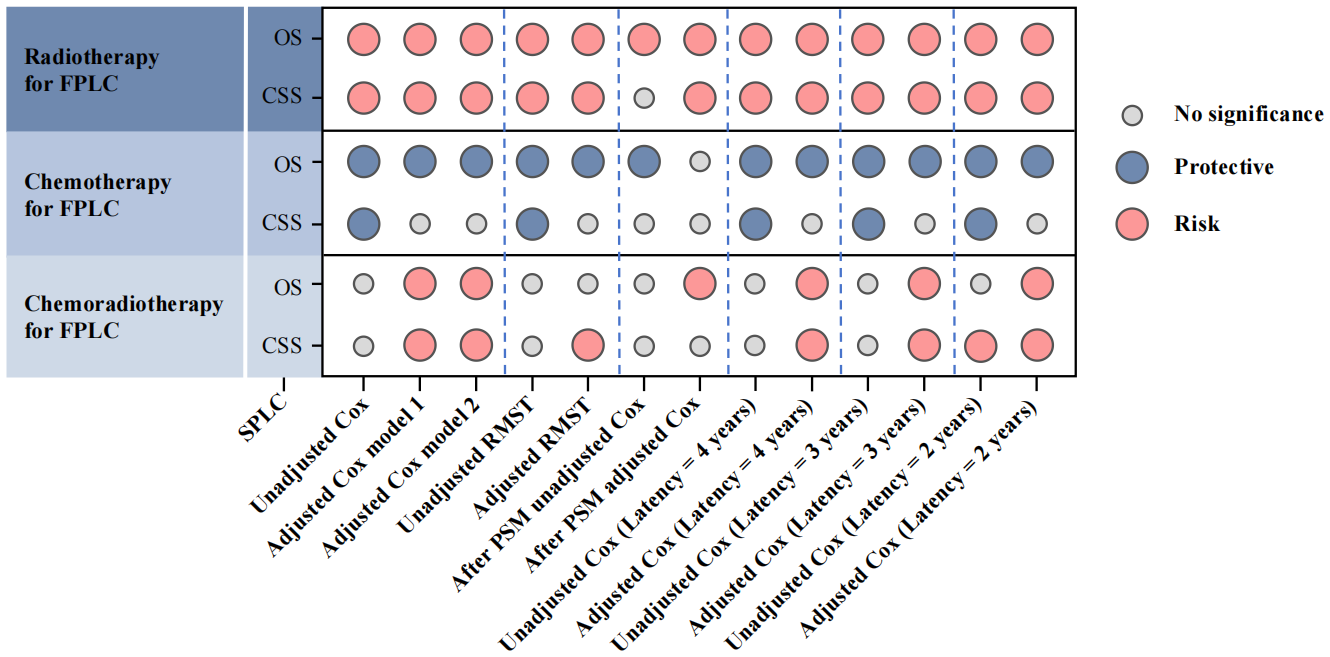
Multiple model results to evaluate the association between different treatments for FPLC and SPLC prognosis. FPLC, first primary lung cancer; SPLC, second primary lung cancer; RMST, restricted mean survival time; OS, overall survival; CSS, cancer-specific survival.

Following previous reports, Cox replication analyses of SPLC prognosis were conducted under varying definitions of SPLC. Multivariate models with 2-, 3-, and 4-year latencies yielded results consistent with those obtained using a 5-year latency (**Supplementary Tables S20**–**S25**). When integrating multiple correction models, overall findings remained consistent with the primary analysis: a history of FPLC radiotherapy and chemoradiotherapy was negatively associated with OS and CSS in SPLC patients, while a history of FPLC chemotherapy conferred a protective effect on OS in this population (**Figure 3**).

### 3.6 Subgroup analysis of SPLC prognosis

Stratified analyses were performed to calculate HR for FPLC treatments across various SPLC populations and identify interactions between FPLC treatments and subgroup variables. Notable interaction effects were observed in CSS between FPLC chemotherapy and latency, as well as between FPLC chemotherapy and SPLC chemotherapy. Additional interaction effects were detected regarding OS and CSS between FPLC chemotherapy and the FPLC pathological subtype, along with interactions between FPLC chemoradiotherapy and the type of FPLC surgery (**Supplementary Figures S2**–**S7**, **Supplementary Tables S26**–**S28**).

To evaluate the differential impact of various FPLC treatment histories on SPLC prognosis, distinct control groups were established. Comparisons revealed that, relative to FPLC chemotherapy history, FPLC radiotherapy (OS: HR = 1.383, 95% CI = 1.151-1.660, *P* < 0.001; CSS: HR = 1.290, 95% CI = 1.046-1.591, *P* = 0.017) and FPLC chemoradiotherapy (OS: HR = 1.354, 95% CI = 1.149-1.596, *P* < 0.001; CSS: HR = 1.353, 95% CI = 1.123-1.631, *P* = 0.002) were linked to poorer prognoses. However, no significant difference was found between the effects of FPLC radiotherapy and FPLC chemoradiotherapy on OS and CSS in SPLC patients (**Table 2**; **Supplementary Table S29**).

**Table 2:** Intra-group comparison of survival for SPLC in patients with different therapies for FPLC.

Variables	OS		CSS	
	Adjusted HR (95%CI)	*P value*	Adjusted HR (95%CI)	*P value*
Chemoradiotherapy of FPLC				
No chemotherapy and radiotherapy	1.000 (Reference)		1.000 (Reference)	
Only chemotherapy	0.849 (0.757 ~ 0.952)	0.005	0.879 (0.771 ~ 1.001)	0.052
Only radiotherapy	1.174 (1.010 ~ 1.365)	0.037	1.134 (0.953 ~ 1.349)	0.157
Chemotherapy and radiotherapy	1.150 (1.008 ~ 1.311)	0.037	1.189 (1.024 ~ 1.380)	0.023
Chemoradiotherapy of FPLC*				
Only chemotherapy	1.000 (Reference)		1.000 (Reference)	
Only radiotherapy	1.383 (1.151 ~ 1.660)	<.001	1.290 (1.046 ~ 1.591)	0.017
Chemotherapy and radiotherapy	1.354 (1.149 ~ 1.596)	<.001	1.353 (1.123 ~ 1.631)	0.002
Chemoradiotherapy of FPLC**				
Only radiotherapy	1.000 (Reference)		1.000 (Reference)	
Chemotherapy and radiotherapy	0.979 (0.810 ~ 1.184)	0.829	1.049 (0.843 ~ 1.304)	0.670

Cox proportional hazards model was used to calculate the HRs and 95%CIs of SPLC survival in FPLC patients receiving different treatments. Controls were replaced under the same analysis, * indicates that the reference group was only FPLC chemotherapy, and ** indicates that the reference group was only FPLC radiotherapy.

### 3.7 Treatment value of re-chemoradiotherapy for SPLC

**Figure 4:**
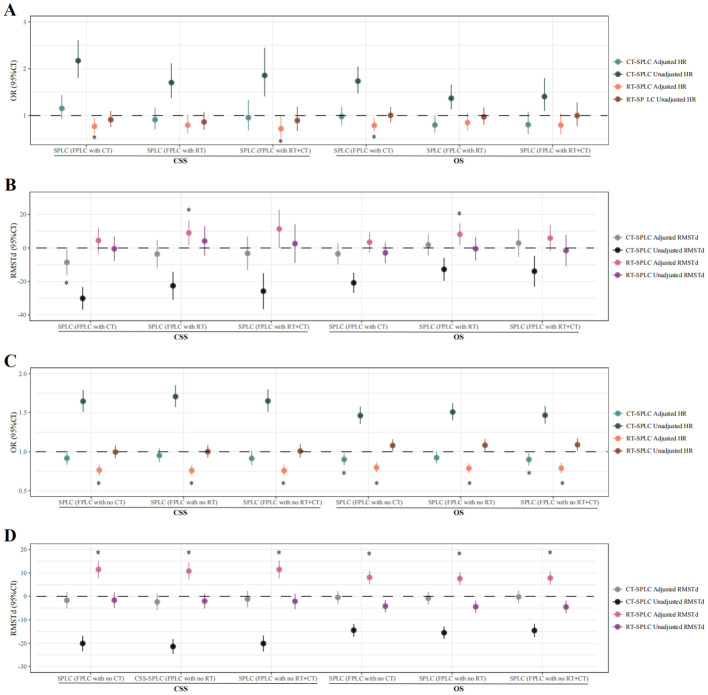
Therapeutic value of radiotherapy and chemotherapy in SPLC patients treated with different FPLC therapies. Cox proportional hazards model and restricted mean survival time analysis were used to calculate the HR, RMSTd, and 95% CI. **(A)**, Cox proportional risk model was used to evaluate the therapeutic value of radiotherapy and chemotherapy in SPLC patients treated with radiotherapy/chemotherapy/chemoradiotherapy for FPLC. **(B)**, Restricted mean survival time analysis was used to evaluate the therapeutic value of radiotherapy and chemotherapy in SPLC patients treated with radiotherapy/chemotherapy/chemoradiotherapy for FPLC. **(C)**, Cox proportional risk model was used to evaluate the therapeutic value of radiotherapy and chemotherapy in SPLC patients treated without radiotherapy/chemotherapy/chemoradiotherapy for FPLC. **(D)**, Restricted mean survival time analysis was used to evaluate the therapeutic value of radiotherapy and chemotherapy in SPLC patients treated without radiotherapy/chemotherapy/chemoradiotherapy for FPLC. * indicates *P*-values of less than 0.05 in multivariate analysis. FPLC, first primary lung cancer; SPLC, second primary lung cancer; HR, hazard ratio; RMSTd, restricted mean survival time difference; CI, confidence interval; OS, overall survival; CSS, cancer-specific survival; RT, radiotherapy; CT, chemotherapy.

To determine whether re-chemoradiotherapy confers survival benefits to SPLC patients with a history of FPLC chemoradiotherapy, evaluations were conducted using Cox regression and RMST analyses. Cox regression findings indicated that re-irradiation improved prognosis in SPLC patients with prior FPLC chemotherapy (OS: HR = 0.789, 95% CI = 0.653–0.953, *P* = 0.014; CSS: HR = 0.770, 95% CI = 0.623–0.951, *P* = 0.016) and reduced the risk of decreased CSS in those with a history of FPLC chemoradiotherapy (HR = 0.719, 95% CI = 0.523–0.989, *P* = 0.042). RMST results showed that re-irradiation increased mean survival time for SPLC patients with a history of FPLC radiotherapy (OS: RMST difference = 8.143 months, 95% CI = 1.657–14.628, *P* = 0.014; CSS: RMST difference = 8.998 months, 95% CI = 1.483–16.513, *P* = 0.019), whereas re-chemotherapy led to a decrease in mean CSS time for those with a history of FPLC chemotherapy (RMST difference = -8.593 months, 95% CI = -16.308 to -0.877, *P* = 0.029; **Figures 4A, B**; **Supplementary Tables S30**–**S35**).

The Cox proportional hazards model indicated that, among SPLC patients without a history of FPLC chemoradiotherapy, irradiation mitigated the risk of declines in both OS and CSS. Conversely, for patients lacking histories of both FPLC chemotherapy and FPLC chemoradiotherapy, re-chemotherapy was associated with favorable OS outcomes (no FPLC chemotherapy history: HR = 0.901, 95% CI = 0.827–0.981, *P* = 0.016; no FPLC chemoradiotherapy history: HR = 0.900, 95% CI = 0.824–0.983, *P* = 0.019) (**Figures 4C, D**; **Supplementary Tables S36**–**S41**).

## 4 Discussion

To the best of our knowledge, this is the first large-scale population-based study to examine the relationship between FPLC chemoradiotherapy and the prognosis, risk, and sensitivity to further chemoradiotherapy in SPLC patients. Prior studies primarily focused on the effects of FPLC radiotherapy on second tumors, neglecting the influence of FPLC chemotherapy history on SPLC and the combined effects of chemoradiotherapy. Moreover, they overlooked the potential benefits of re-chemoradiotherapy for the SPLC population (16, 20). Our study addresses these gaps, concluding that while FPLC treatment with surgery, radiotherapy, and chemotherapy does not increase the risk of SPLC, it significantly alters SPLC survival outcomes and sensitivity to chemoradiotherapy. Specifically, a history of FPLC radiotherapy serves as an independent risk factor for OS and CSS in SPLC patients, whereas a history of FPLC chemotherapy confers a protective effect on OS. Notably, SPLC patients with both FPLC radiotherapy and chemotherapy histories still exhibit poorer prognoses. Furthermore, re-radiotherapy can enhance survival in SPLC patients with a history of FPLC chemoradiotherapy, although this benefit is more pronounced in those without such a history. For SPLC patients with prior FPLC chemotherapy, average CSS time is reduced, while chemotherapy improves OS in patients without a history of FPLC chemotherapy.

Generally, SPLC diagnosed more than 1 year after FPLC is classified as metachronous SPLC, whereas lung malignancies identified at the initial FPLC diagnosis or within 12 months are termed synchronous SPLC (21, 22). Distinguishing SPLC from FPLC recurrence is critical, as misidentification can lead to inappropriate treatment and biased prognostic evaluations. According to Martini’s criteria, SPLC with a latency period of less than two years must display distinct pathological types from FPLC (9). With the advent of various adjuvant therapies, histological transformations in lung cancer have become increasingly common. Non-small cell lung cancer may transform into small cell lung cancer (23), and similarly, lung adenocarcinoma can evolve into squamous carcinoma as a mechanism of drug resistance (24). This indicates potential biases in SPLC definitions based solely on pathological differences. Exclusively excluding patients with SPLC whose pathology differs from FPLC not only reduces the sample size, leading to decreased statistical power, but also fails to substantially address errors in defining SPLC. Despite genomic methods that can help differentiate between recurrence and second primary cancers (25, 26), their application is significantly limited within the SEER database, which only includes detailed clinical baseline features of cancer patients without any genomic biomarker data. Therefore, our study design faces significant challenges in tumor definition disturbances. Our study reduced the error caused by SPLC definition problems through various sensitivity analyses to improve statistical power, and included subjects as much as possible to reveal our findings. Firstly, we applied a stricter definition for the target FPLC population, consisting of non-advanced stage patients who underwent curative surgical resection, significantly reducing the possibility of FPLC recurrence bias. Additionally, the diagnosed SPLC patients had an interval of at least five years from FPLC diagnosis, providing a sufficient time window to reduce the association between the two occurrences of lung cancer. We also performed replicated analyses for SPLC defined with 2-year, 3-year, and 4-year intervals to assess whether diagnostic interval differences truly affect SPLC definition. Notably, conclusions across different diagnostic intervals were consistent, largely confirming the accuracy of SEER data in defining second primary tumors. Thirdly, in evaluating the impact of previous FPLC treatments on SPLC prognosis, we employed analytical models both including and excluding FPLC patient characteristics, yielding consistent conclusions which further affirm that SPLC occurrence is unlikely to be significantly influenced by the included FPLC features. Fourthly, we balanced the baseline patient data through PSM, ensuring that even if FPLC recurrence affects SPLC definition, the bias would likely be consistent across both groups, thus maintaining the reliability of our conclusions. Finally, it is important to note that FPLC patients undergoing chemotherapy and radiotherapy may represent a more advanced tumor stage. If tumor recurrence itself muddles the SPLC definition, then both FPLC radiotherapy and chemotherapy would be associated with a poorer SPLC prognosis. However, our results indicate that the effects of FPLC chemotherapy and radiotherapy on SPLC prognosis are polarized, which does not align with the hypothetical scenario of FPLC recurrence being misidentified as SPLC, and partially corroborates previous reports (16). These outcomes suggest that the likelihood of FPLC recurrence blending into our SPLC cohort is very low.

Previous research has demonstrated that children who survive cancer for more than five years often face a broad spectrum of adverse health outcomes due to therapeutic exposures, including secondary malignancies (27). In the St. Jude Lifetime (SJLIFE) cohort, which included 3,006 childhood cancer survivors, the prevalence of pathogenic and likely pathogenic (PLP) germline variants in 60 well-defined cancer predisposition genes was 5.8%, approximately ten times the rate of 0.6% observed in non-cancer controls. *RB1*, *NF1*, and *BRCA2* were the most frequently affected genes (28), and this prospective study found that PLP carriers without prior radiation exposure have an increased likelihood of developing any type of tumor subsequently, while those with prior radiation exposure show a heightened risk of developing breast cancer and sarcoma. Additionally, subsequent integrated cohort data revealed that carriers of PLP variants also have an increased risk of mortality due to subsequent malignancies (29). However, the impact of specific prior treatments, such as FPLC chemoradiotherapy, on the occurrence and prognosis of subsequent tumors has not been individually assessed. Although FPLC treatment exposure appears to have a minimal impact on the incidence of SPLC, our findings suggest it may alter the prognosis and sensitivity to re-treatment in SPLC cases. These observations, based on aggregate population data, do not take into account genetic information, thereby limiting our understanding of the interactions between genetic factors and treatment exposures. Subgroup analysis based on clinical characteristics has shown variances in SPLC outcomes depending on the pathology type, surgical modality of FPLC, latency periods, and chemotherapy regimens, suggesting that genetic background may influence treatment exposure interactions. Therefore, future genomic studies focusing on the different treatment histories of SPLC patient populations are necessary, especially for FPLC treatment exposures. Such studies could elucidate how specific genetic variations interact with treatment histories, affecting disease progression and aiding in more precise disease prognosis predictions, ultimately providing tailored treatment strategies.

This study’s strengths include a substantial sample size drawn from the SEER database, robust sensitivity analyses, and a well-structured design. However, several limitations warrant consideration. First, although the SEER database provides a broad, population-based sample, it lacks detailed information on specific chemotherapy and radiotherapy protocols, such as dosage, modality, and administration frequency. This limits our ability to analyze the impacts of different treatment regimens in depth. If more comprehensive FPLC treatment data were available in the future, it would allow for more precise insights. Second, although we aimed to control for potential biases in defining SPLC, our definition may still fall short of fully capturing real-world scenarios. Implementing genomic standards for SPLC identification could improve accuracy and strengthen future findings. Third, lifestyle factors, including diet, physical activity, and comorbidities, likely influence SPLC onset and prognosis. The absence of lifestyle data in the SEER database limits our ability to perform a comprehensive multifactorial analysis, including PSM, potentially overlooking lifestyle-related variables. Future studies that include lifestyle factors may help clarify their impact on SPLC risk and prognosis.

Despite these limitations, our findings offer practical clinical insights. By identifying factors potentially associated with SPLC prognosis, our results can guide clinicians in long-term surveillance and treatment strategies for SPLC patients. For instance, in clinical practice, when encountering an SPLC patient, clinicians should inquire about prior treatments received for the initial FPLC, particularly whether radiotherapy or chemotherapy were used. If the patient underwent radiotherapy as part of their FPLC treatment, our findings suggest that they may have a poorer prognosis for SPLC. This could inform decisions regarding the need for additional treatments such as further radiotherapy, which might be considered to improve their prognosis and guide follow-up care. Additionally, our study suggests that treatment dosage may influence SPLC prognosis. Future research that incorporates genomic criteria to refine SPLC identification could lead to a more precise understanding of SPLC progression and provide a foundation for tailored patient care.

In summary, this cohort study employed a multi-faceted approach to evaluate the impact of FPLC radiotherapy and chemotherapy history on the risk, prognosis, and sensitivity to subsequent radiotherapy and chemotherapy in SPLC patients. The findings offer valuable insights for prognostic evaluation and treatment selection in individuals developing SPLC after FPLC.

## Data Availability

The original contributions presented in the study are included in the article/**Supplementary Material**. Further inquiries can be directed to the corresponding author/s.
