# Supplementary material for: Impact of chemoradiotherapy for first primary lung cancer on the prognosis and re-chemoradiotherapy sensitivity of second primary lung cancer
Source: Front Immunol. 2025 Jan 27;16:1492501. doi: 10.3389/fimmu.2025.1492501 (PMC11808144; doi:10.3389/fimmu.2025.1492501)
Supplement: Supplementary file 2 [file Table1.docx]

**Table 1: Risk of developing SPLC in patients with different therapies for FPLC.**

| **Variables** | **SPLC risk** | | | | |
| --- | --- | --- | --- | --- | --- |
|  | **Unadjusted HR (95%CI)** | ***P value*** |  | **Adjusted HR (95%CI)** | ***P value*** |
| Radiotherapy of FPLC |  |  |  |  |  |
| No | 1.000 (Reference) |  |  | 1.000 (Reference) |  |
| Yes | 1.026 (0.943 ~ 1.116) | 0.550 |  | 1.031 (0.939 ~ 1.131) | 0.520 |
| Chemotherapy of FPLC |  |  |  |  |  |
| No | 1.000 (Reference) |  |  | 1.000 (Reference) |  |
| Yes | 1.102 (1.029 ~ 1.181) | 0.006 |  | 0.999 (0.924 ~ 1.079) | 0.980 |
| Chemoradiotherapy of FPLC |  |  |  |  |  |
| No chemotherapy and radiotherapy | 1.000 (Reference) |  |  | 1.000 (Reference) |  |
| Only chemotherapy | 1.094 (1.004 ~ 1.191) | 0.040 |  | 0.977 (0.895 ~ 1.067) | 0.610 |
| Only radiotherapy | 0.940 (0.823 ~ 1.074) | 0.360 |  | 0.978 (0.854 ~ 1.120) | 0.750 |
| Chemotherapy and radiotherapy | 1.107 (0.997 ~ 1.229) | 0.057 |  | 1.059 (0.952 ~ 1.178) | 0.290 |
| Chemoradiotherapy of FPLC* |  |  |  |  |  |
| Only chemotherapy | 1.000 (Reference) |  |  | 1.000 (Reference) |  |
| Only radiotherapy | 0.860 (0.738 ~ 1.001) | 0.051 |  | 1.001 (0.856 ~ 1.170) | 0.990 |
| Chemotherapy and radiotherapy | 1.012 (0.890 ~ 1.150) | 0.860 |  | 1.083 (0.952 ~ 1.233) | 0.230 |
| Chemoradiotherapy of FPLC** |  |  |  |  |  |
| Only radiotherapy | 1.000 (Reference) |  |  | 1.000 (Reference) |  |
| Chemotherapy and radiotherapy | 1.177 (0.999 ~ 1.387) | 0.051 |  | 1.082 (0.917 ~ 1.277) | 0.084 |

Footnote: Fine-Gray competitive risk model was used to calculate the HRs and 95%CIs of SPLC risk in FPLC patients receiving different treatments. Controls were replaced under the same analysis, * indicates that the reference group was only FPLC chemotherapy, and ** indicates that the reference group was only FPLC radiotherapy.
