# Supplementary material for: Impact of chemoradiotherapy for first primary lung cancer on the prognosis and re-chemoradiotherapy sensitivity of second primary lung cancer
Source: Front Immunol. 2025 Jan 27;16:1492501. doi: 10.3389/fimmu.2025.1492501 (PMC11808144; doi:10.3389/fimmu.2025.1492501)
Supplement: Supplementary file 3 [file Table2.docx]

**Table 2: Intra-group comparison of survival for SPLC in patients with different therapies for FPLC.**

| **Variables** | **OS** | |  | **CSS** | |
| --- | --- | --- | --- | --- | --- |
|  | **Adjusted HR (95%CI)** | ***P value*** |  | **Adjusted HR (95%CI)** | ***P value*** |
| Chemoradiotherapy of FPLC |  |  |  |  |  |
| No chemotherapy and radiotherapy | 1.000 (Reference) |  |  | 1.000 (Reference) |  |
| Only chemotherapy | 0.849 (0.757 ~ 0.952) | 0.005 |  | 0.879 (0.771 ~ 1.001) | 0.052 |
| Only radiotherapy | 1.174 (1.010 ~ 1.365) | 0.037 |  | 1.134 (0.953 ~ 1.349) | 0.157 |
| Chemotherapy and radiotherapy | 1.150 (1.008 ~ 1.311) | 0.037 |  | 1.189 (1.024 ~ 1.380) | 0.023 |
| Chemoradiotherapy of FPLC* |  |  |  |  |  |
| Only chemotherapy | 1.000 (Reference) |  |  | 1.000 (Reference) |  |
| Only radiotherapy | 1.383 (1.151 ~ 1.660) | <.001 |  | 1.290 (1.046 ~ 1.591) | 0.017 |
| Chemotherapy and radiotherapy | 1.354 (1.149 ~ 1.596) | <.001 |  | 1.353 (1.123 ~ 1.631) | 0.002 |
| Chemoradiotherapy of FPLC** |  |  |  |  |  |
| Only radiotherapy | 1.000 (Reference) |  |  | 1.000 (Reference) |  |
| Chemotherapy and radiotherapy | 0.979 (0.810 ~ 1.184) | 0.829 |  | 1.049 (0.843 ~ 1.304) | 0.670 |

Footnote: Cox proportional hazards model was used to calculate the HRs and 95%CIs of SPLC survival in FPLC patients receiving different treatments. Controls were replaced under the same analysis, * indicates that the reference group was only FPLC chemotherapy, and ** indicates that the reference group was only FPLC radiotherapy.
