# Supplementary material for: Impact of chemoradiotherapy for first primary lung cancer on the prognosis and re-chemoradiotherapy sensitivity of second primary lung cancer
Source: Front Immunol. 2025 Jan 27;16:1492501. doi: 10.3389/fimmu.2025.1492501 (PMC11808144; doi:10.3389/fimmu.2025.1492501)

**Supplementary Figure Legends**

**Supplementary Figure 1. Comparison between the characteristics of four statistical methods.**

HR: Hazard ratio; RMTLd: the difference in restricted mean time lost; RMSTd: restricted mean survival time difference.

**Supplementary Figure 2. Subgroup analysis of survival for SPLC in patients with radiotherapy for FPLC (OS).**

FPLC: first primary lung cancer; SPLC: second primary lung cancer; OS: overall survival; HR: Hazard ratio; CI: confidence Interval.

FPLC radiotherapy for reference group, FPLC radiotherapy for intervention group.

**Supplementary Figure 3. Subgroup analysis of survival for SPLC in patients with radiotherapy for FPLC (CSS).**

FPLC: first primary lung cancer; SPLC: second primary lung cancer; CSS: cancer-specific survival; HR: Hazard ratio; CI: confidence Interval.

FPLC radiotherapy for reference group, FPLC radiotherapy for intervention group.

**Supplementary Figure 4. Subgroup analysis of survival for SPLC in patients with chemotherapy for FPLC (OS).**

FPLC: first primary lung cancer; SPLC: second primary lung cancer; OS: overall survival; HR: Hazard ratio; CI: confidence Interval.

FPLC chemotherapy for reference group, FPLC chemotherapy for intervention group.

**Supplementary Figure 5. Subgroup analysis of survival for SPLC in patients with chemotherapy for FPLC (CSS).**

FPLC: first primary lung cancer; SPLC: second primary lung cancer; CSS: cancer-specific survival; HR: Hazard ratio; CI: confidence Interval.

FPLC chemotherapy for reference group, FPLC chemotherapy for intervention group.

**Supplementary Figure 6. Subgroup analysis of survival for SPLC in patients with chemoradiotherapy for FPLC (OS).**

FPLC: first primary lung cancer; SPLC: second primary lung cancer; OS: overall survival; HR: Hazard ratio; CI: confidence Interval.

FPLC chemoradiotherapy for reference group, FPLC chemoradiotherapy for intervention group.

**Supplementary Figure 7. Subgroup analysis of survival for SPLC in patients with chemoradiotherapy for FPLC (CSS).**

FPLC: first primary lung cancer; SPLC: second primary lung cancer; CSS: cancer-specific survival; HR: Hazard ratio; CI: confidence Interval.

FPLC chemoradiotherapy for reference group, FPLC chemoradiotherapy for intervention group.

**Supplementary Figure 1.**

**
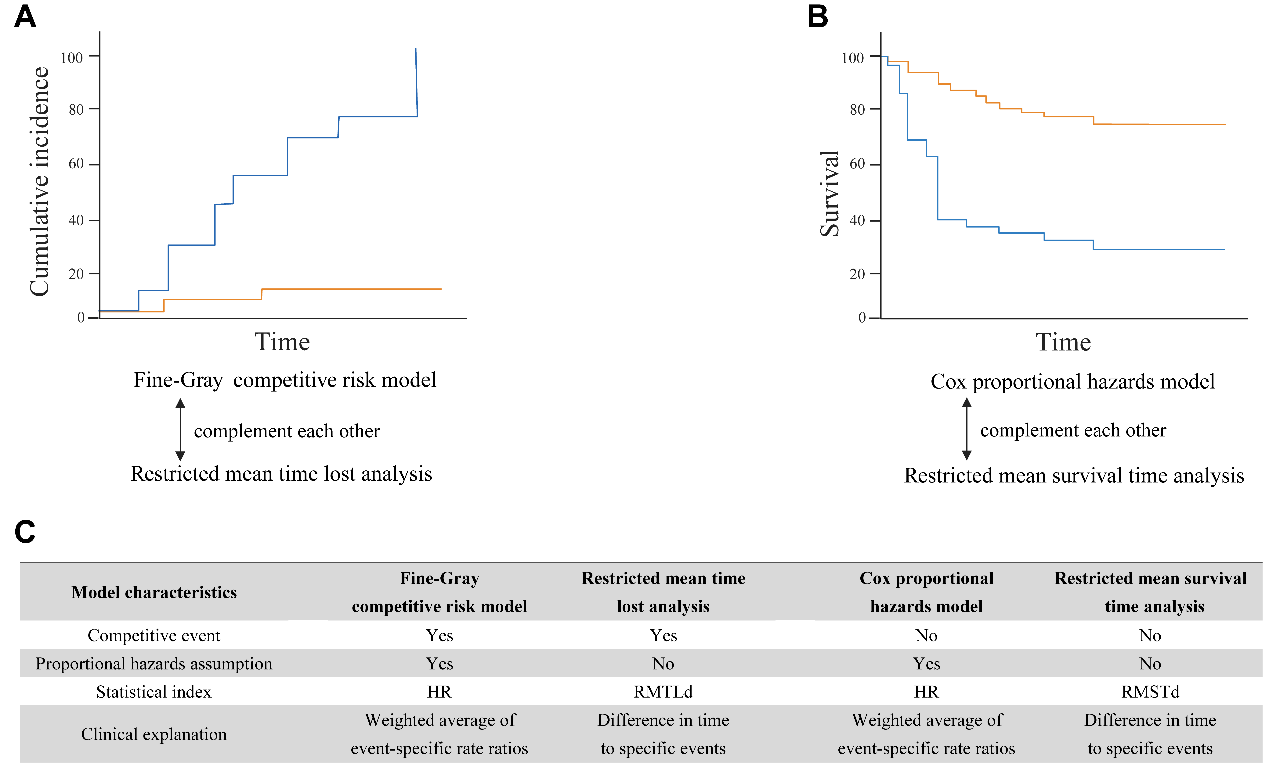
**

**Supplementary Figure 2. Subgroup analysis of survival for SPLC in patients with radiotherapy for FPLC (OS).**

FPLC: first primary lung cancer; SPLC: second primary lung cancer; OS: overall survival; HR: Hazard ratio; CI: confidence Interval.

FPLC radiotherapy for reference group, FPLC radiotherapy for intervention group.


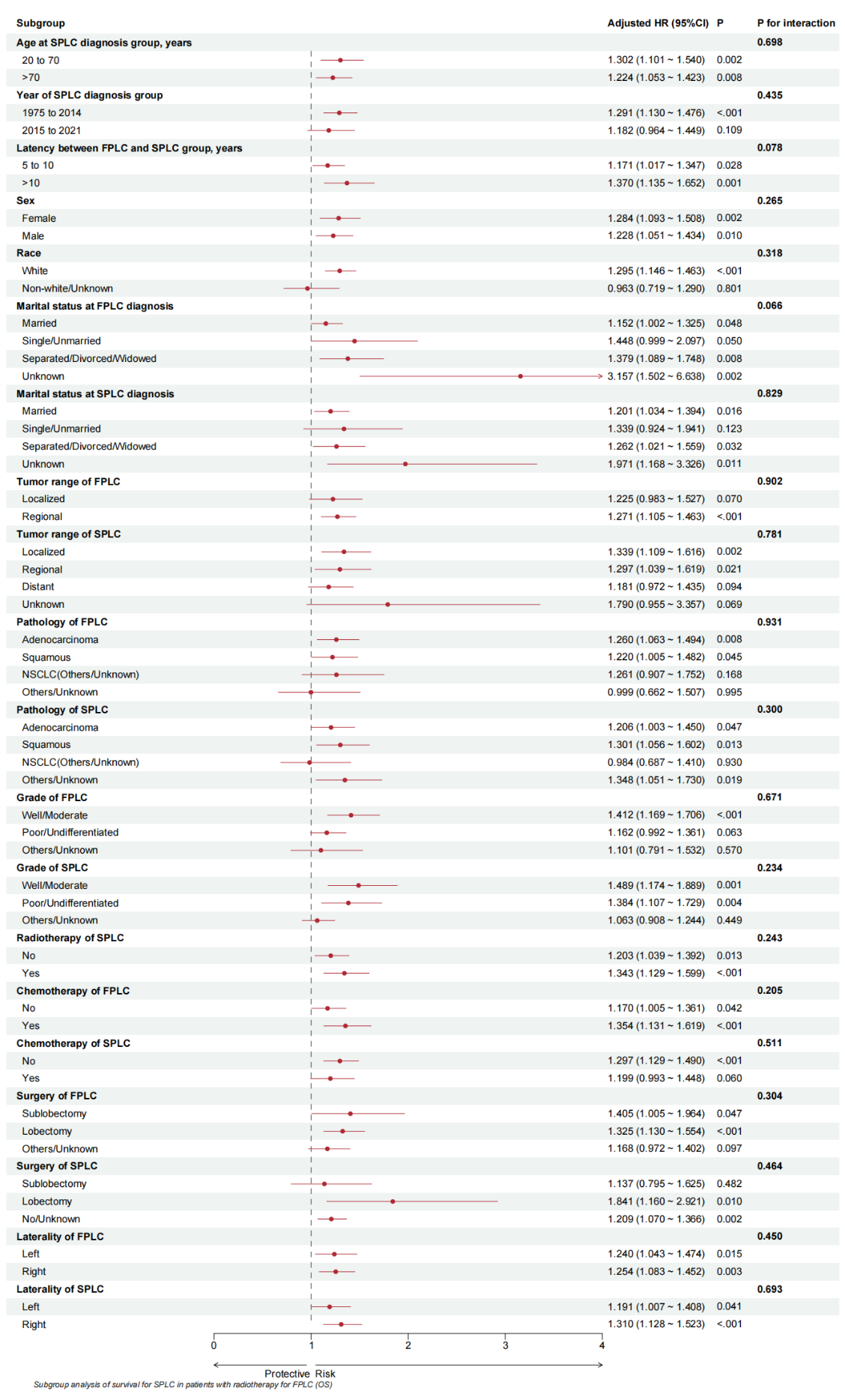


**Supplementary Figure 3. Subgroup analysis of survival for SPLC in patients with radiotherapy for FPLC (CSS).**

FPLC: first primary lung cancer; SPLC: second primary lung cancer; CSS: cancer-specific survival; HR: Hazard ratio; CI: confidence Interval.

FPLC radiotherapy for reference group, FPLC radiotherapy for intervention group.


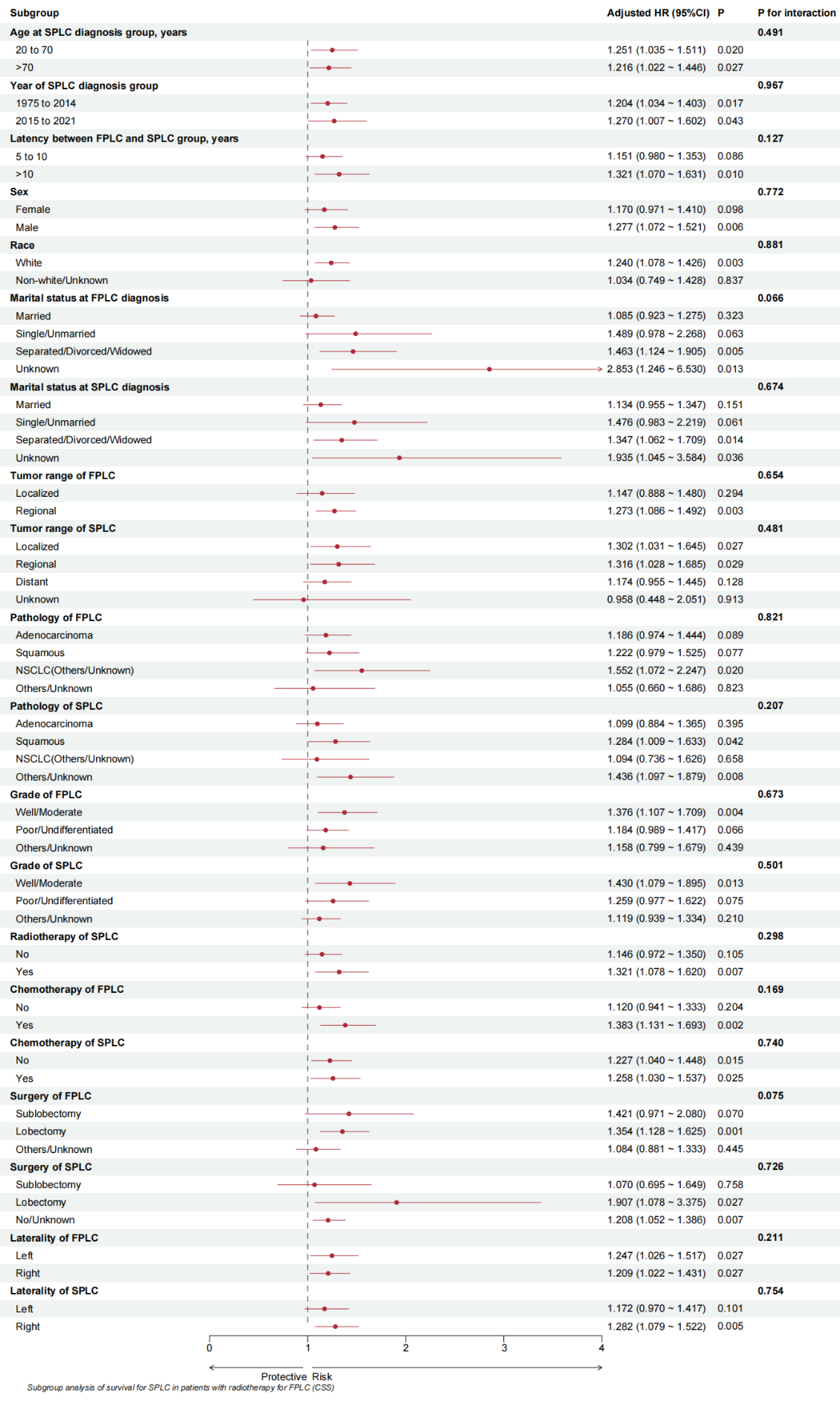


**Supplementary Figure 4. Subgroup analysis of survival for SPLC in patients with chemotherapy for FPLC (OS).**

FPLC: first primary lung cancer; SPLC: second primary lung cancer; OS: overall survival; HR: Hazard ratio; CI: confidence Interval.

FPLC chemotherapy for reference group, FPLC chemotherapy for intervention group.


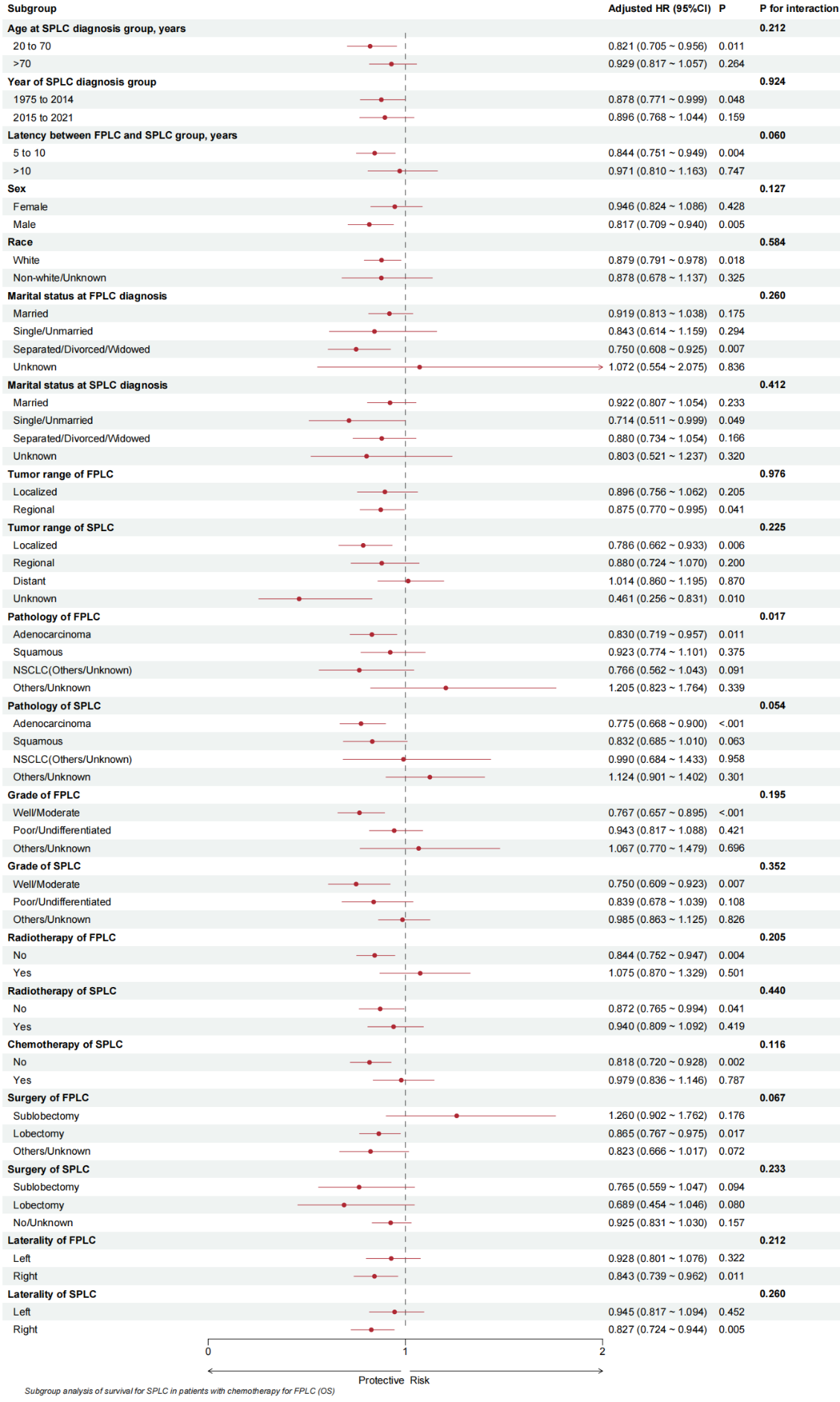


**Supplementary Figure 5. Subgroup analysis of survival for SPLC in patients with chemotherapy for FPLC (CSS).**

FPLC: first primary lung cancer; SPLC: second primary lung cancer; CSS: cancer-specific survival; HR: Hazard ratio; CI: confidence Interval.

FPLC chemotherapy for reference group, FPLC chemotherapy for intervention group.


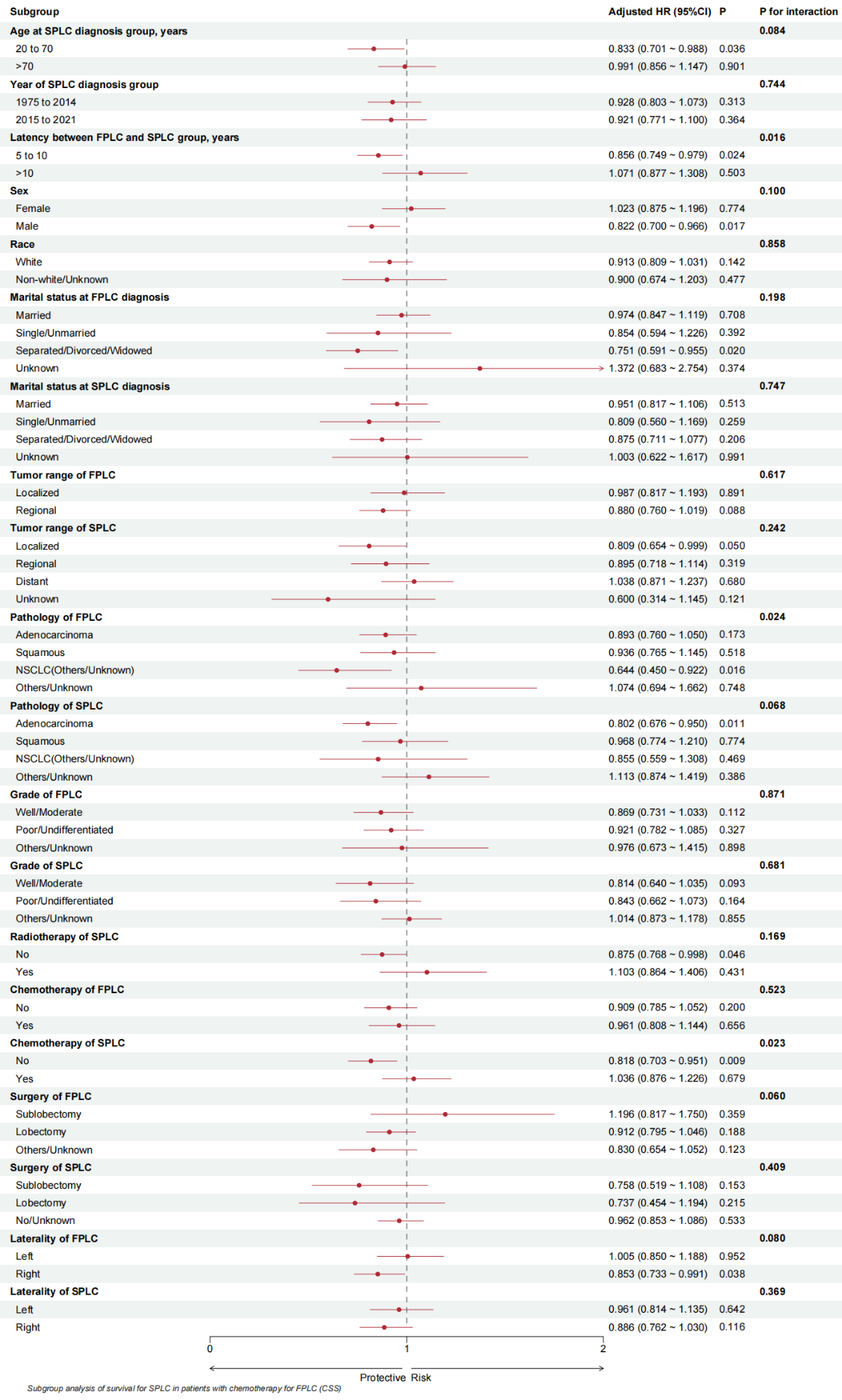


**Supplementary Figure 6. Subgroup analysis of survival for SPLC in patients with chemoradiotherapy for FPLC (OS).**

FPLC: first primary lung cancer; SPLC: second primary lung cancer; OS: overall survival; HR: Hazard ratio; CI: confidence Interval.

FPLC chemoradiotherapy for reference group, FPLC chemoradiotherapy for intervention group.


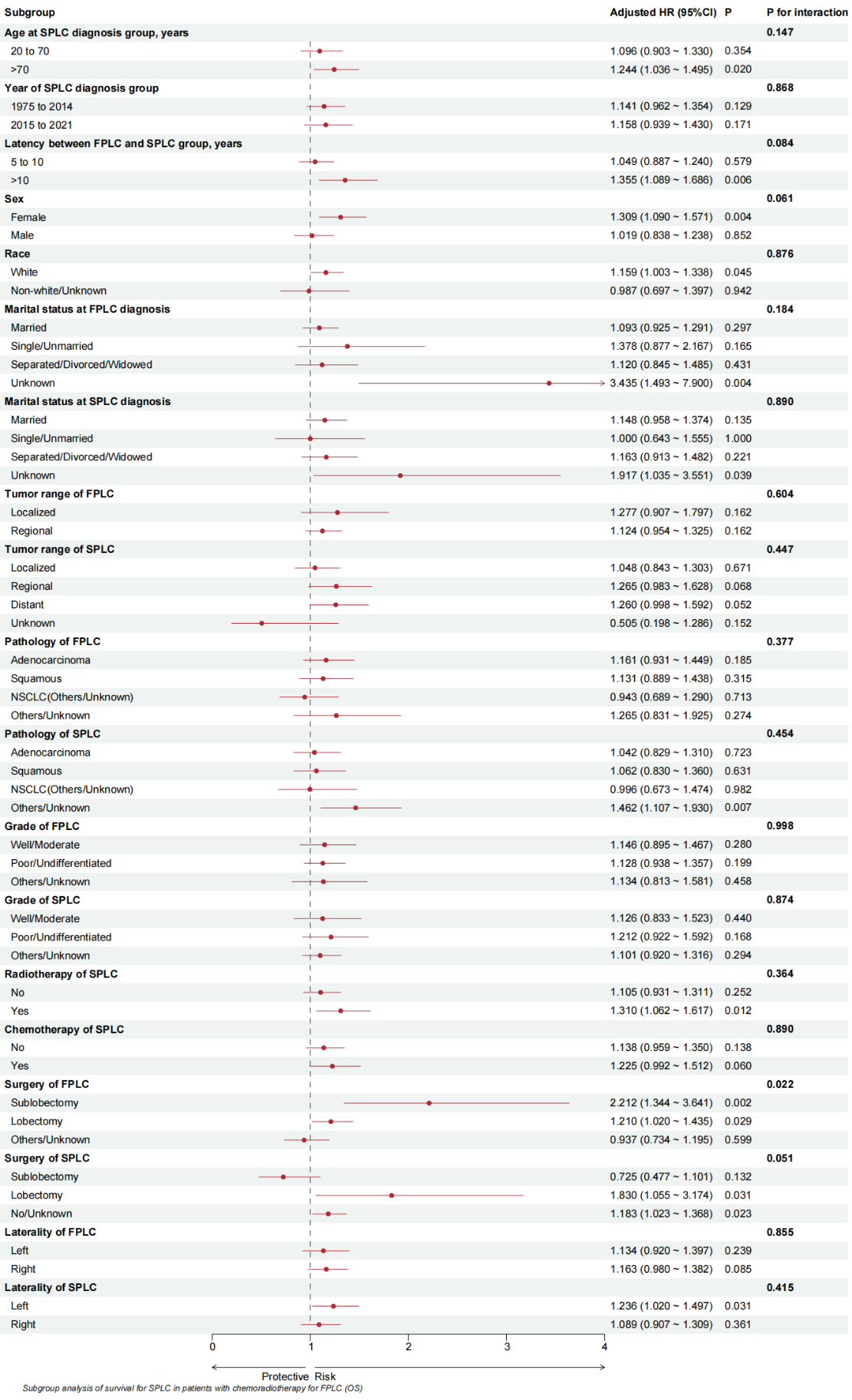


**Supplementary Figure 7. Subgroup analysis of survival for SPLC in patients with chemoradiotherapy for FPLC (CSS).**

FPLC: first primary lung cancer; SPLC: second primary lung cancer; CSS: cancer-specific survival; HR: Hazard ratio; CI: confidence Interval.

FPLC chemoradiotherapy for reference group, FPLC chemoradiotherapy for intervention group.


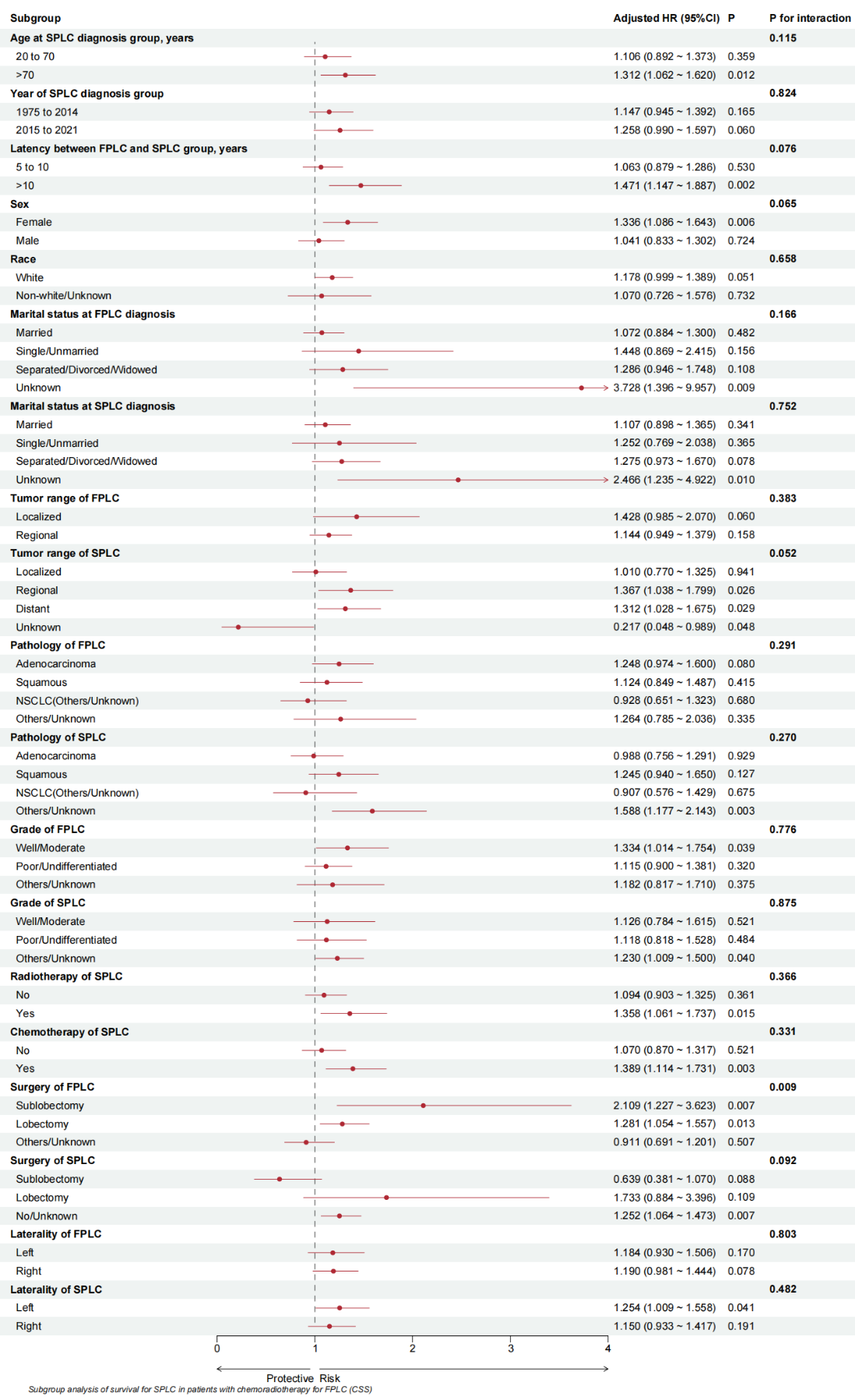

Supplement: Supplementary file 4 [file Table3.docx]
